# Formulating the best *Helbat*: A Tigraian semi-liquid fasting condiment

**DOI:** 10.1016/j.heliyon.2023.e17114

**Published:** 2023-06-09

**Authors:** Solomon Fitsum, Goitom Gebreyohannes Berhe, Desta Berhe Sbhatu, Gebreselema Gebreyohannes

**Affiliations:** Mekelle University, PO Box 1632/231, Mekelle, Ethiopia

**Keywords:** *Helbat*, *Megulat* variety, Mineral composition, Proximate analysis, Sensory evaluation

## Abstract

Helbat (hl'bət), a fasting semi-liquid condiment, is a popular indigenous traditional fermented product in Tigrai (a.k.a. Tigray), Ethiopia. It is formulated using *Vicia faba* flour prepared from mildly roasted dry beans. Despite its dietary, nutritional, religious, and cultural significance, the condiment is yet not exposed to scientific study. Hence, this research was carried out to: (a) ascertain the best faba bean variety for preparing high quality *Helbat*, (b) develop the formulation and preparation protocol of high quality *Helbat*, (c) determine the effects of fermentation and storage temperature and time on the physicochemical characteristics, proximate and mineral compositions, microbiological properties, and sensory qualities of *Helbat*, and (d) determine the shelf life of *Helbat* as the function of storage time and temperature. To this end, three faba bean varieties used in preparing *Helbat* as well as fermenting and stored *Helbat* products were studied using standard physicochemical, microbiological, and sensory evaluation procedures. Quantitative data were processed using inferential statistical methods and mean (±SD) values were compared at *a priori* set *p*-value of ≤0.05. The study showed that: (a) the best faba bean variety for making high quality *Helbat* was *Megulat*; (b) the best *Helbat* formulation constituted 400 wt units of bean flour, 7 wt units of garlic, 6 wt units of ginger, 5 wt units fenugreek, 5 wt units of corrorima, 8 wt units of red pepper, and 3 wt units of black mustard; (c) the best *Helbat* formulation was nutritionally rich to supplement fasting consumers with proteins, fats, and minerals; (d) the *Helbat* formulation remained safe for up to eight weeks while maintaining its attractive sensory qualities when stored at 11–15 °C, and (e) increasing the fermentation and storage times led to changes in its physicochemical properties (i.e., temperature, pH, total titratable acidity, and total soluble solute) and depletion of many nutritionally vital components such as fats, proteins, and minerals. Thus, unless production and storage conditions are somehow modified, *Helbat* needs to be consumed fresh after three to seven days of fermentation. But further research may be required to make this recommendation conclusively. Exploring into its antioxidant properties and lactic acid bacteria (LAB) may highlight its qualities further.

## 1 Introduction

Fermentation is a handy, indigenous biotechnology in preparing fermented foods, beverages, and dairy products in all civilizations and communities of the world [1]. Such biotechnologies are common in many Ethiopian traditions and cultures in producing foods and beverages [2]. Preparation of traditional fermented foods and beverages are based on indigenous knowledge systems and technologies [3]. Such knowledge systems and technologies often evolve into the traditions and cultures of the communities involved. Fermented foods and condiments play vital roles in supplementing human dietary and nutritional requirements and protecting against many infectious diseases. They also have outstanding traditional and cultural significance and economic contributions.

The rich cultural diversity rendered Ethiopia to become home to several traditional fermented beverages (e.g., *Tella* (*Suwa* in Tigrinya), *Taj* (*Mies* in Tigrinya), *Araqi*, *Borde*, *Shamita*, and *Kineto*), foods (e.g., *Injera*, *Dabo* (*Gogo* in Tigrinya), *Ambasha* (*Hembasha* in Tigrinya), *Qotcho*, and *Bulla*), condiments (e.g., *Siljo*, *Helbat*, *Awaze*, and *Datta*), and dairy products (e.g., *Ergo*, (*Rig'o* in Tigrinya)) [1,2]. These traditional fermented products are very popular in urban and rural communities [1,4]. They are important part of livelihood and economic activities of hundreds of thousands of households throughout the country [2,5].

Tigrai (northern Ethiopia), is home to many fermented foods, beverages, condiments, and dairy products that are part and parcel of the local traditions and cultures. *Injera* (soft and thin pancake), *Hembasha* (thick, flat and circular bread), and *Gogo* or *Guaguba* (round bread) are key fermented staple foods. *Suwa* (Tigraian ale), *Mies* (Tigraian honey wine), *Araqi*, and *Korefie* are principal traditional alcoholic beverages. They are very vital products for livelihood and economic activities for several thousand households. Likewise, *Helbat*, *Siljo*, *Hazo*, *Awaze*, *Dilikh*, and *Senafitch* are common fermented condiments. *Helbat*, *Siljo*, and *Hazo* are unique to Tigrai. *Rig'o* (yoghurt) and *Ajibo* (cheese) are also fermented traditional dairy products in Tigrai.

Condiments such as *Helbat* and *Siljo* are also important dietary and nutritional components during fasting seasons among the Tigraian Orthodox Christian households. *Helbat* is the most favored condiment prepared from flours of faba beans (*Vicia faba*) and field peas (*Pisum sativum*) [6] enriched with many spices including garlic (*Allium sativum*), ginger (*Zingiber officinale* Rose L), fenugreek (*Trigonella foenum graecum*), chili pepper (*Capsicum annum*), Ethiopian cardamom (*Aframomum corrorima*), and black mustard (*Brassica nigra*). The flours of the bean and peas are prepared from mildly roasted grains. *Helbat* is a good source of proteins, carbohydrates, fats, and minerals [6]. It is served as cold sauce with *Tihlo* and *Injera*− the most popular dishes in Tigrai.

Unfortunately, preparations of high quality indigenous beverages, foods, condiments, and dairy products are not easy. They require knowledgeable, skillful, and experienced natives. And yet, with politico-cultural, socio-economic, and scientific-technological changes, such traditional knowledge and technologies are not effectively passing into new generations of natives. Efforts towards the characterization of the indigenous food and beverage products and standardizations of their formulations and preparations are highly recommended as they open opportunities for modernization of their production. In line with these rationales, this study aims to achieve four goals; namely (a) ascertaining the best faba bean variety for preparing high quality *Helbat*, (b) developing formulation and preparation protocols of high quality *Helbat*, (c) determining the effects of fermentation and storage time and temperature on the physicochemical characteristics, proximate and mineral compositions, microbiological properties and sensory qualities of *Helbat*, and (d) determining the shelf life of *Helbat* as the function of storage temperature and time. The findings of the study are useful in assisting consumers in making proper dietary and nutritional choices and entrepreneurs aspiring to open businesses on *Helbat* production and supply. Natives interested in preparing *Helbat* for household consumption are also provided with a handy and clear formulation procedure. Furthermore, it has put a good deal of data for researchers to make further studies on the product.

## 2 Materials and methods

### 2.1 Collection of ingredients for preparing *Helbat*

**Fig. 1:**
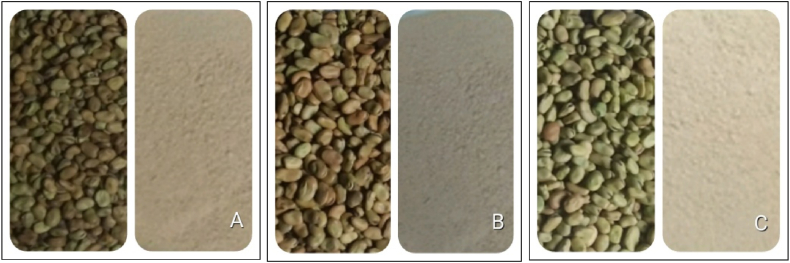
Grains and flours of bean varieties used in the study (A: *Gora*; B: *Moti*; C: *Megulat*).

**Fig. 2:**
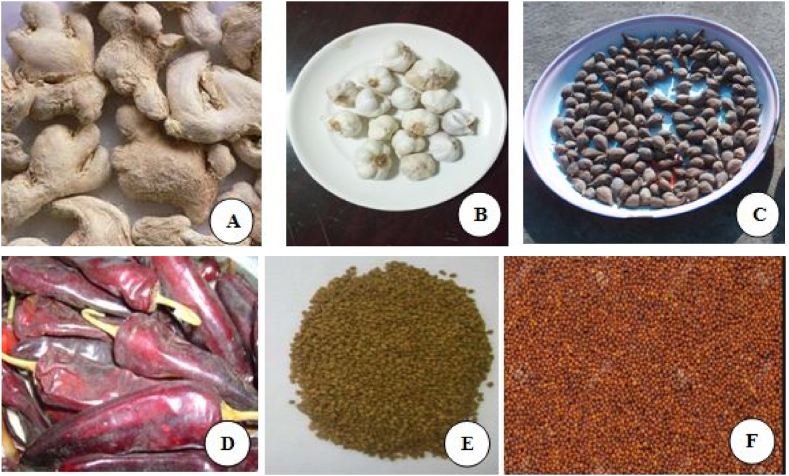
Spices used in the study (A: Ginger; B: Garlic; C: Corrorima; D: Chili Pepper; E: Fenugreek; F: Black Mustard).

Three *Vicia faba* varieties; namely, *Gora* (go'ræ), Megulat (m'gʊ’læt), and Moti (mo'tɪ) were selected to prepare *Helbat* and study their nutritional, physicochemical, and microbiological characteristics and sensory qualities (Fig. 1A–C). The varieties were collected from Mekelle Agricultural Research Center, Mekelle city, Northern Ethiopia. Spice additives, including garlic (*Allium sativum*) (rose variety), ginger (*Zingiber officinale* Rose L), and fenugreek (*Trigonella foenum graecum*) (Challa variety) were acquired from Tigrai Agricultural Research Institute, Mekelle city. Other spice additives; namely, chili pepper (*Capsicum annum*) (Mareko fana variety), Ethiopian cardamom (*Aframomum corrorima*) (local variety) and black mustard (*Brassica nigra*) (local variety) (Fig. 2A–F) were purchased from a local market called Mayda Agame, in Adigrat city, Tigrai, Northern Ethiopia [7]. The seeds and spice additives were fresh, viable, and disease-free. All ingredients were cleaned off any dirt, sorted, and washed with distilled water as appropriate, and sun-dried.

### 2.2 Research laboratories

*Helbat* products were prepared from flours of three faba bean varieties in a stone and brick room (LWH: 4.0 m × 5.0 m × 3.5 m) with wooden door and window set up for the purpose. The room was emptied, cleaned, and fumigated, and furnished with sterilized utensils used for preparing the *Helbat*. The preparations of the products were carried out at ambient temperature. The products were also allowed to ferment in this room. Analyses of proximate composition and mineral contents of the beans and the *Helbat* products were conducted in the geochemical lab of the College of Natural and Computational Sciences at Mekelle University, Mekelle city. Physicochemical analyses of the beans and the *Helbat* products were carried out in the tissue culture laboratory of Tigrai Biotechnology Center Pvt. Ltd. Co., Mekelle. Microbiological studies were carried out in the tissue culture laboratory of Tigrai Biotechnology Center and microbiology laboratories of the colleges of Veterinary Medicine and Health Sciences, Mekelle University. Storage and shelf life evaluation were carried out in the Tigrai Biotechnology Center.

### 2.3 Establishing *Helbat* formulation protocol

The formulations of *Helbat* condiments were prepared by mixing 2 kg (2000 g) of flour from each variety and different proportions of spice additives. The flours of the three bean varieties and various proportions of spice additives (also referred to as ingredients) were decided in consultation with native women locally recognized as reputable *Helbat*-makers. All the formulations had 2,000 g (92.166%) bean flour and 170 g (7.834%) spice additives. This proportion is equivalent to 400 wt units of bean flour to 34 wt units of spice additives (Table 1).

**Table 1:** Different proportions of ingredients for preparing the best *Helbat*.

Formulations	Spice additives in gram (weight units)							
	Faba beans	Garlic	Ginger	Fenugreek	Corrorima	Chili Pepper	Mustard	Total
F1	2000 (400)	45 (9)	20 (4)	15 (3)	35 (7)	50 (10)	5 (1)	2000 (434)
F2	2000 (400)	40 (8)	25 (5)	20 (4)	30 (6)	45 (9)	10 (2)	2000 (434)
F3	2000 (400)	35 (7)	30 (6)	25 (5)	25 (5)	40 (8)	15 (3)	2000 (434)
F4	2000 (400)	30 (6)	35 (7)	30 (6)	20 (4)	35 (7)	20 (4)	2000 (434)
F5	2000 (400)	25 (5)	40 (8)	35 (7)	15 (3)	30 (6)	25 (5)	2000 (434)

### 2.4 *Helbat* preparation

Faba bean grains were cleaned by removing any dirt and gravel, sorted by removing all damaged grains, washed using tap water three times, and sun-dried for one day by spreading on clean polyethylene plastic sheet. Then, they were moderately roasted in metal pan and crushed with stone mill to remove the seed coats and split the grains into cotyledons. The split grains were sun-dried for two days by spreading on clean polyethylene plastic sheet. The sun-dried grains were, then, milled into flours, sieved using 1 mm sieve, and packed in dry, clean polyethylene bags. Two (2) kg flour was added into 3.50 L boiling water in clay pot, and further boiled for 90 min. The content was homogenized by continuously stirring using clean wooden rod. It was left for 24 h to cool into cold paste and pressed through 2 mm mesh to remove any un-homogenized content. All the spice additives were, likewise cleaned, processed by grinding and/or mashing, and required amounts were added to the cold paste as per the formulation protocol given in Table 1. The content of each formulation was further homogenized with clean wooden rod.

### 2.5 Tests and experiments of the study

Traditionally, *Helbat* is prepared from various bean varieties and its fermentation is carried out in clean clay pot tightly sealed with plastic sheet tied around its mouth using a rope. It is often ready for consumption after three to six days of fermentation at ambient temperature. But the fermentation can go for additional days, and the product can be stored for several weeks. Thus, physicochemical and microbial studies as well as sensory evaluation procedures were carried out to select the best bean variety, establish the best formulation, and ascertain the best fermentation time. Once the best formulation was established, physicochemical properties, proximate and mineral compositions, microbial properties, and sensory qualities of the *Helbat* product made as per the formulation protocol were examined during fermentation and storage by taking required samples. To this end, multiple samples of *Helbat* products were subjected to one to seven days of fermentation at ambient temperature. They were also stored for several weeks at four storage temperature ranges.

### 2.6 Analysis of physicochemical properties

The pH, total titratable acidity (TTA), and total soluble solutes (TSS) of flours of the beans and fermenting and stored *Helbat* products were determined according to the methods established in the manual of the Association of Analytical Chemists (i.e., 925.05) (AOAC) [8]. (a) Determination of pH was carried by taking 10 g sample (of flour and fermenting and stored *Helbat*), mixing it with 100 mL distilled water in a beaker, and homogenizing the mixture by stirring for 5 min. The pH meter was calibrated at 7:00 with buffer solution and measurements were carried out in triplicate. (b) The TTA was determined by taking 5 mL sample, mixing it with 40 mL distilled water, adding 2 to 3 drops of phenolphthalein to it as indicator, and titrating the solution against 0.1 N NaOH solution till the solution turned pink. TTA is calculated as: TTA (%) = [Titrant × Equivalent Weight of Ascorbic Acid × 100] ÷ [Volume of Sample Taken × 1000]. (c) The TSS/°Brix levels of the beans and the *Helbat* formulations were determined using a portable digital refractometer (ATAGO model). Samples were equilibrated at 20 °C before measurements were carried out. One (1) mL of each sample was poured onto a refractometer prism and readings were recorded.

### 2.7 Analysis of proximate composition

Proximate compositions (moisture, ash, total carbohydrate, crude protein, and crude fat contents and gross energy) of the bean varieties, and the fermenting and stored *Helbat* products were determined as per the method described in the manual of the Association of Analytical Chemists (i.e., 925.05) (AOAC) [8] and other relevant standard procedures [9,10].

#### 2.7.1 Determination of moisture content

It was determined by oven drying method. Twenty five (25) gram sample was weighed in clean porcelain crucible and heated to 100 °C. The crucible was cooled and weighed. Then, the crucible with the sample was heated in an electric oven for ca. 6 hrs at 100 °C. It was then cooled in desiccator and weighed again. The moisture content in the sample was calculated as: % Moisture Content = [(W_1_–W_2_) ÷ (W_1_)] × 100; where W_1_ is weight of original sample and W_2_ is weight of sample after drying.

#### 2.7.2 Determination of ash content

Ash content was determined by putting 3 g sample into a clean porcelain crucible, heating it at 100 °C, and cooling and weighing the crucible and its contents. The crucible was then placed in a furnace for ca. 4 h at ca. 600 °C, cooled in desiccator, and weighed. Complete ashing was assured by further heating the crucible and its content in the furnace for 30 min, cooled, and weighed again. This was repeated until consecutive weights became the same and the ash turned white. Percentage ash content was calculated as: %Ash = [[(W_C+A_) − (W_C_)] ÷ [(W_C+S_) − (W_C_)]] × 100; where W_C_ refers to weight of crucible, W_A_ refers to weight of ash, and W_S_ refers to weight of sample.

#### 2.7.3 Determination of crude fat content

Crude fat content was analyzed by manual extraction method [9]. A 0.40 g sample (W_1_) was transferred into 250 mL flask and 2 mL alcohol was added to it. The content was stirred to moisten and mix it completely with 10 mL diluted 4 N HCl. The flask was set on heater and refluxed for 30 min, stirred at frequent intervals until the sample was completely hydrolyzed, and 10 mL alcohol was added to it and cooled. The flask was rinsed and its content was poured into extraction tube with 25 mL diethyl ether in three portions. The tube was closed with cork and shaken vigorously for 1 min. Then, 25 mL petroleum ether was added to the extraction tube and was shaken vigorously for 1 min until the upper portion of the liquid appeared clear. The ether-fat solution was filtered through a funnel firmly plugged with cotton in its stem part. The solution was allowed to free the passage of ether into the flask. The flask was dried in oven at 100 ± 5 °C, cooled in desiccator, and weighed (W_2_). The extraction of the liquid sample was repeated twice in the tube using the same solvent. The clear ether solutions were transferred through the same funnel into the same flask. The solvents were allowed to evaporate completely on water bath at 70–80 °C. The fat was dried in oven at 100 ± 5 °C until constant weight was obtained. Finally, the flask was cooled in desiccator and weighed (W_3_). Crude fat content (%) was calculated as: % Crude Fat = [(W_3_ − W_2_) ÷ (W_1_)] × 100; where W_1_ is weight of sample, W_2_ is weight of dried flak before extraction, and W_3_ is weight dried flask after extraction.

#### 2.7.4 Determination of crude fiber content

Crude fiber content was determined by putting 2 g sample (W_1_) in a round bottom flask. Then, 100 mL 0.023 M 1.25% sulfuric acid solution was added to it and the mixture was boiled under reflux for 30 min. The hot solution was filtered from the insoluble matter, washed with acid-free hot water, and transferred into conical flask. Then, 100 mL hot (0.312 M) NaOH solution was added, boiled under reflux for 30 min, and the content was quickly filtered. The residue was weighed, washed with acetone, put in crucible and dried to constant weight in oven, cooled in desiccator, and weighed with the crucible (W_2_). The crucible with its content was incinerated in muffle furnace at 555 °C for 2 h, cooled in desiccator, and reweighed (W_3_). The crude fiber content was determined as: % Crude Fiber = [(W_2_– W_3_) ÷ (W_1_)] × 100; where W_1_ is weight of sample, W_2_ is weight of insoluble matter, and W_3_ is weight of ash.

#### 2.7.5 Determination of crude protein content

Crude protein content was determined as per the methods described in the works of Kruis (2014) [10]. Determination of total nitrogen was carried out by taking 2 g sample into digestion bomb. Then, 3 mL H_2_O_2_ and 2.5 mL digestion mixture (comprising sulfuric acid, salicylic acid, and selenium) was added to the digestion bomb, and the content was heated in dry oven at 100 °C for 12 h. The content was diluted with 15 mL distilled water and filtered using filter paper into 50 mL flask. Finally, n1 and n2 nitrogen powders were added for nitrogen determination and mixed for 10 min. Total nitrogen was read by UV spectrometer. The crude protein content was calculated as: % Crude Protein = Total Nitrogen (mg/100 g) × 6.25 (Conversion Factor).

#### 2.7.6 Determination of total carbohydrate content

The total carbohydrate contents of the samples were determined using the exclusion method by subtracting the proportions of all components of the proximate analyses from 100%. It was calculated as: % Carbohydrate = 100 − (% Moisture + % Ash + % Crude Fat + % + Crude Fiber + Crude Protein).

#### 2.7.7 Determination of gross energy

Gross energy values (kcal/100 g) of the samples were estimated by multiplying the sum of crude protein contents and total carbohydrate by 4 and adding it to the crude fat content multiplied by 9 (i.e., Gross Energy Value (in kcal/100 g) = [(% Crude Protein Content + % Total Carbohydrate) × 4] + [(% Crude Fat Content) × 9]).

### 2.8 Mineral analysis

Mineral analyses were conducted using the method described by Kruis (2014) [10]. Magnesium (Mg), calcium (Ca), zinc (Zn), copper (Cu), and iron (Fe) contents of samples of digested beans and fermenting and stored *Helbat* products were determined by Atomic Absorption Spectrometer (VARIANN model). Sodium (Na) and potassium (K) contents were determined using Flame Photometer (JENWAY model). Likewise, nitrogen (N) and phosphorus (P) contents were determined using the UV Light Photometer. In all cases, the contents were expressed in mg/L.

### 2.9 Microbial analyses

The microbial profiles the fermenting and stored *Helbat* products were determined at 24 h of interval during the seven days fermentation as well as on the 15th, 30th, 45th, and 55th days during storage to examine their microbial safety. For this purpose, 1 g *Helbat* was dissolved in 9 mL sterile distilled water, shaken for 1 min, and the mixture was subjected to serial dilution to generate 10^−6^ mg/mL mixture [11].

#### 2.9.1 Yeast and mold count

Yeast and mold count was determined by taking 1 mL sample from the 10^−6^ diluted sample solution, inoculating it onto yeast peptose dextrose (YPD) broth and potato dextrose agar (PDA) medium supplemented with 60 mg/L chloramphenicol using spread plating technique. The plates were incubated at 25 °C for 5 days and visible colonies were counted and expressed as total yeast and mold in colony-forming units per gram (CFU/g) of samples [12].

#### 2.9.2 Total bacterial count

Total bacterial count was determined by taking 0.1 mL serially diluted 0.1% (w/v) sample, inoculating it onto standard plate count agar (PCA) (Oxoid), and incubating it at 30–32 °C for 48 h. The colonies were counted using the colony counter [11]. Total coliforms were enumerated by taking 0.1 mL serially diluted sample solution and inoculating it onto MacConkey broth, *Salmonella*, and *Shigella* agar (SSA), Eosin methylene agar (EMBA), Tryptone soy agar (TSA) (Oxoid) after 30 min overlays with violate red bail agar (VRBA) medium. The plates were incubated at 37 °C for 24 h. Then, the colonies were counted using the colony counter [12].

#### 2.9.3 Load of lactic acid bacteria

The load LAB was determined by taking 0.1 mL serially diluted sample and spread plating on pre-dried surfaces of MRS agar plates. The plates were incubated at 30–32 °C for 48 h and the colonies were counted [11]. The MRS agar medium and broth were also used as selective media for isolation and purification of the LAB. Thus, diluted samples were poured, plated on MRS agar plates, and incubated in anaerobic jar at 30–32 °C for 72 h. Typical colonies on MRS agar were sub-cultured twice overnight in MRS broth and subsequently transferred into MRS agar for purity [12]. The isolates were classified to genus level based on morphological, biochemical, and physiological characteristics [13].

### 2.10 Sensory evaluation of *Helbat*

Sensory evaluations of the *Helbat* products were carried out multiple times by 30 experienced participants (20 men, 10 women; age 35–75 years old) purposefully selected from the community. Whereas 26 (87%) of the participants were primary school complete, 4 (13%) were without any formal schooling. All of them have agreed to participate in the sensory evaluation by signing informed consent. Thus, they were designated as judges and were trained on how to judge and rate the qualities of the products. The sensory evaluations of the products were based on five attributes; namely, ‘color’, ‘taste’, ‘aroma’, ‘mouth feel’, and ‘overall acceptability’ based on the works of Ahmad et al. (2015) [14]. The attributes were evaluated using a 9-point Hedonic scale where ‘9’ stands for ‘like extremely’, ‘8’ stands for ‘like very much’, ‘7’ stands for ‘like moderately’, ‘6’ stands for ‘like slightly’, ‘5’ for ‘neither like nor dislike’, ‘4’ stands for ‘dislike slightly’, ‘3’ stands for ‘dislike moderately’, ‘2’ stands for ‘dislike very much’, and ‘1’ stands for ‘dislike extremely’.

### 2.11 Determination of shelf life of *Helbat*

Shelf life of *Helbat* was determined by storing it for up to 55 days at different storage temperatures and evaluating its sensory qualities and its ability to inhibit the growth of spoiling microorganism. The storage temperatures were <10 °C (1), 11–15 °C (2), 16–20 °C (3), 21–25 °C (4), and ambient temperature (control). Thus, the shelf life of *Helbat* was determined by sensory evaluation and through observation of the products for growth of spoiling microorganisms.

### 2.12 Data sources and analyses

Data were collected from all observations (including measurements and counts), tests, and experiments. All sampling, measurements, and tests (experiments) were conducted in triplicate. Quantitative data were analyzed using relevant descriptive and inferential statistical methods with SPSS Version 20 software. Inferential (sample) data were analyzed using the analysis of variance (ANOVA) at *a priori* set *p-*value of ≤0.05. Post-hoc comparisons of means were carried out using Least Significance Difference (LSD). Qualitative data collected by visual and microscopic observations were used to strengthen the results of the quantitative data analyses.

## 3 Results and discussion

### 3.1 Selecting the best *Vicia faba* variety for making the best *Helbat*

#### 3.1.1 Proximate compositions of the Vicia faba varieties

The proximate analysis of the flours of the three bean varieties showed that most of the mean percentage compositions of their constituents were statistically significantly variable (*p* ≤ 0.05; Table 2). Whereas the *Gora* variety yielded statistically higher moisture content (3.94 ± 0.01%), ash content (13.30 ± 0.30%), and crude fiber (12.83 ± 0.76%), the *Megulat* variety resulted in higher crude fat (6.88 ± 0.08%) and crude protein (31.02 ± 0.07%). The *Moti* variety, on the other hand, has higher total carbohydrate (47.19 ± 0.62%) and energy (329.45 ± 2.05 Kcal/100 g). An earlier study on the proximate compositions of three Ethiopian dry bean varieties called *Roba*, *Tabor*, and *Awash* showed higher mean moisture content (9.08–11.06%), lower mean ash content (3.52–4.26%), higher mean carbohydrate content (56.52%–61.53%), lower mean crude fiber content (4.69–5.53%), lower mean crude protein (19.62–27.55%), and lower mean crude fat content (1.25–2.44%) [15]. As *Helbat* is a fasting condiment replacing meat and dairy products, its fat and protein content makes it nutritionally preferred. This test shows that the *Mugulat* variety can be the preferred one for making *Helbat*.

**Table 2:** Proximate compositions of the three faba bean varieties.

Proximate analysis	Mean (±SD) proximate composition (%)		
	Gora	Megulat	Moti
Moisture content	3.94 ± 0.01^a^	3.78 ± 0.02^b^	1.93 ± 0.03^c^
Ash content	13.30 ± 0.30^a^	12.63 ± 0.35^b^	8.36 ± 0.04^c^
Crude fat	4.20 ± 0.05^b^	6.88 ± 0.08^a^	2.97 ± 0.08^c^
Crude protein	28.42 ± 0.07^b^	31.02 ± 0.07^a^	27.55 ± 0.11^c^
Crude fiber	12.83 ± 0.76^a^	11.68 ± 0.59^a^	10.00 ± 0.50^b^
Total carbohydrate	37.30 ± 0.91^c^	39.22 ± 0.48^b^	47.19 ± 0.62^a^
Energy value (Kcal/100 g)	324.83 ± 3.44^a^	318.73 ± 2.27^b^	329.45 ± 2.05^a^

Means (±SD) in the same row with different letters are statistically significantly different at p ≤ 0.05.

#### 3.1.2 Mineral composition of the Vicia faba varieties

Results of analyses of mineral composition of flours of the varieties revealed statistically significantly variable mean contents in many cases. Whereas *Gora* has the highest mean Ca and Fe content, *Megulat* has highest mean Mg, K, Na, P, N, Zn, and Cu contents. *Moti* has the second highest mean Mg and Ca contents (Table 3). These results imply that the mineral-rich bean of *Megulat* variety is preferred for fasting periods to supplement nutrient limitations.

**Table 3:** Mineral composition of the three faba bean varieties.

Minerals	Mean (±SD) mineral composition (mg/L)		
	Gora	Megulat	Moti
Magnesium	590.00 ± 1.00^a^	630.00 ± 2.64^a^	616.60 ± 2.08^a^
Calcium	720.00 ± 1.00^a^	640.00 ± 2.64^c^	680.00 ± 1.00^b^
Potassium	1982.30 ± 0.15^b^	2008.40 ± 0.64^c^	1947.30 ± 0.05^a^
Sodium	36.30 ± 0.05^b^	37.40 ± 0.01^c^	24.00 ± 0.10^a^
Phosphorus	476.10 ± 0.02^b^	507.60 ± 0.06^c^	442.60 ± 0.05^a^
Nitrogen	457.60 ± 0.05^a^	463.60 ± 0.28^b^	438.60 ± 0.05^c^
Zinc	17.20 ± 0.01^b^	19.50 ± 0.12^a^	15.90 ± 0.00^b^
Iron	62.60 ± 0.05^c^	49.90 ± 0.07^b^	43.00 ± 0.05^a^
Copper	6.70 ± 0.01^b^	7.80 ± 0.05^a^	6.20 ± 0.01^b^

Means (±SD) in the same row with different letters are statistically significantly different at p ≤ 0.05.

### 3.2 Selecting the best *Helbat* via sensory evaluation

The mean likings of the five *Helbat* formulations of each bean variety have shown statistically significant differences among the formulations and bean varieties (Table 4). Exploring into the results of each variety, Formulation #3 is most liked since it received the highest mean likings in three or more attributes. Even though the differences lacked statistical significance in four of the five attributes, Formulation #3 of *Megulat* variety received the highest mean likings in four attributes (i.e., taste, color, aroma, and overall acceptability) and the second highest in the fifth attribute (mouth feel). Thus, this exercise was instrumental in selecting the best formulation (Formulation #3) and the best variety (*Megulat*). Further studies on the physicochemical and microbiological features of the condiment were carried out using *Megulat Helbat* prepared using Formulation #3. Formulation #3 was composed of 400 wt units of bean flour, 7 wt units of garlic, 6 wt units of ginger, 5 wt units fenugreek, 5 wt units of corrorima, 8 wt units of red pepper, and 3 wt units of black mustard.

**Table 4:** Sensory evaluation of *Helbat* formulations.

Variety	Formulations	Mean (±SD) scores of sensory attributes (Degrees of likings)				
		Taste	Color	Aroma	Mouth feel	Overall acceptance
*Megulat*	F1	3.33 ± 0.33^e^	6.33 ± 0.33^c^	7.33 ± 0.33^b^	4.66 ± 0.33^a-c^	5.66 ± 0.33^f^
	F2	7.33 ± 0.33^b^	6.66 ± 0.33^cb^	8.66 ± 0.33^a^	7.88 ± 0.83 ^a-c^	7.31 ± 0.30^bc^
	F3	8.66 ± 0.33^a^	8.66 ± 0.33^a^	9.00 ± 1.00^a^	8.10 ± 0.50^a^	8.66 ± 0.33^a^
	F4	7.00 ± 1.00^b^	7.22 ± 0.51^b^	7.22 ± 0.69	7.22 ± 0.51^a-d^	7.33 ± 0.33^a^
	F5	5.33 ± 0.33^c^	5.44 ± 0.19^de^	7.33 ± 0.33^b^	5.97 ± 0.36^e^	6.55 ± 0.19
*Gora*	F1	4.33 ± 0.33^d^	4.11 ± 0.19^g^	6.66 ± 0.33^bc^	5.55 ± 0.50^ef^	5.33 ± 0.33^f^
	F2	7.11 ± 0.19^b^	5.55 ± 0.38^de^	7.99 ± 0.33^a^	8.11 ± 0.19^a^	7.11 ± 0.19^b-d^
	F3	8.22 ± 0.19^a^	6.55 ± 0.19^bc^	8.22 ± 0.19^a^	7.99 ± 0.33^ab^	8.55 ± 0.50^a^
	F4	6.55 ± 0.50^b^	6.55 ± 0.50^bc^	6.88 ± 0.19^cb^	6.99 ± 0.33^cd^	7.33 ± 0.33^bc^
	F5	4.88 ± 0.50^d^	5.22 ± 0.19^e^	6.99 ± 0.33^bc^	6.33 ± 0.33^de^	6.33 ± 0.33^e^
*Moti*	F1	3.33 ± 0.33^e^	6.11 ± 0.19^cd^	6.77 ± 0.19^bc^	5.11 ± 0.19^fg^	7.33 ± 0.33^fg^
	F2	6.77 ± 0.19^b^	6.55 ± 0.50^bc^	7.99 ± 0.33^a^	7.22 ± 0.69^a-d^	6.33 ± 0.33^cd^
	F3	8.11 ± 0.19^a^	8.11 ± 0.19	8.11 ± 0.19^a^	7.11 ± 0.19^a-d^	5.11 ± 0.19^b^
	F4	6.99 ± 0.33^b^	7.10 ± 0.50^b^	6.33 ± 0.33^c^	6.44 ± 0.38^de^	6.99 ± 0.33^cd^
	F5	4.99 ± 0.33^cd^	5.44 ± 0.50^de^	5.33 ± 0.33^d^	4.33 ± 0.33^g^	4.55 ± 0.38^g^

Means (±SD) in the same row with different letters are statistically significantly different at p ≤ 0.05.

### 3.3 Characteristics of fresh *Megulat Helbat*

Fresh *Helbat* refers to a product fermented for 1–7 days. *Helbat* is traditionally consumed within a week after preparation, and is regarded as fresh. Below, the characteristics of fresh *Megulat Helbat* prepared as per Formulation #3 are provided.

#### 3.3.1 Physicochemical properties

The study of the physicochemical properties, including temperature, pH, total titratable acidity (TTA), and total dissolved solute (TSS) of the product is important to judge its quality and readiness for consumption. The mean temperature of the *Helbat* product decreased from 27.73 ± 0.41 (in day 1) to 24.00 ± 0.00 (in day 7). The mean variations between consecutive days were statistically significant except between day 5 and day 6 and day 6 and day 7 (*p* ≤ 0.05; Table 5). Such an observation may be because of high biochemical processes involving aerobic microbes during the earlier days of fermentation.

**Table 5:** Physicochemical properties of fermenting *Megulat Helbat*.

Fermentation days	Mean (±SD) values of measurement of physicochemical parameters			
	Temperature (°C)	pH	TTA (%)	TSS (°Brix)
1	27.73 ± 0.41^a^	5.86 ± 0.02^a^	0.15 ± 0.00^g^	12.50 ± 0.36^a^
2	26.26 ± 0.11^b^	5.42 ± 0.02^b^	0.22 ± 0.00^f^	10.83 ± 0.03^b^
3	25.30 ± 0.10^c^	4.84 ± 0.02^c^	0.28 ± 0.00^e^	9.22 ± 0.02^c^
4	24.84 ± 0.06^d^	4.25 ± 0.01^d^	0.35 ± 0.01^d^	8.40 ± 0.02^d^
5	24.63 ± 0.15^ed^	3.96 ± 0.01^e^	0.42 ± 0.01^c^	7.28 ± 0.08^e^
6	24.40 ± 0.20^e^	3.91 ± 0.01^f^	0.55 ± 0.03^b^	6.76 ± 0.05^f^
7	24.00 ± 0.00^f^	3.84 ± 0.01^g^	0.64 ± 0.01^a^	6.51 ± 0.10^f^

Means (±SD) in the same column with different letters are statistically significantly different at p ≤ 0.05.

The mean pH of *Helbat* decreased with increasing the days of fermentation. It decreased from 5.86 ± 0.02 in day 1–3.84 ± 0.01 in day 7. The decrement in pH from day to day was statistically significant (*p* ≤ 0.05; Table 5). Similar results were observed in other Ethiopian traditional condiments known as *Siljo*, *Awaze*, and *Hazo*. The mean pH of *Siljo* decreased from 6.0 to 3.9 within eight days of fermentation [6]. *Siljo*, prepared from beans, is a closely related product to *Helbat*. Likewise, whereas the mean pH of *Awaze* decreased from 5.3 to 3.78 in 13 days fermentation [16], the mean pH of *Hazo* was decreased from 5.8 to 3.81 in six days [11].

The mean TTA of *Helbat*, on the other hand, increased from day 1 to day 7 with statistical significance in each passing day. It increased from 0.15 ± 0.00 (in day 1) to 0.64% (in day 7) (*p* ≤ 0.05; Table 5). Similar patterns were observed in *Siljo* [6], *Hazo* [11], and a third Ethiopian traditional condiment called *Datta* [15] – a similar product to *Awaze*. The increment in mean TTA in *Siljo* was from 0.36 to 0.75% in eight days, while that of *Hazo* was from 0.06 to 0.36% in six days. Likewise, the mean TTA increment in *Datta* was from 0.04 to 0.14% in 13 days.

The mean TSS of the *Megulat Helbat* product decreased from 12.50 °Brix in day 1–6.51 °Brix in day 7. The decrement in mean TSS between each passing day was statistically significant, except between day 6 and day 7 (*p* ≤ 0.05; Table 5). The decrease in mean TSS as well as the decrease in mean pH and corresponding increase in mean TTA might be due to the dominance of the fermentation environment by lactic acid bacteria (LAB) that degrade carbohydrates and leave fibers like pectin and similar products [17] while making the *Helbat* more acidic.

#### 3.3.2 Proximate composition

*Megulat Helbat* product was left for 7-day fermentation and the changes in its proximate composition were analyzed in days 1, 3, 5, and 7. With the exception of energy value, the mean percentage compositions of all constituents showed changes during the days of fermentation with many of the changes in mean values being statistically significant (*p* ≤ 0.05; Table 6). Moisture content of the product increased from 7.03 ± 0.03 (in day 1) to 11.02 ± 0.05 (in day 7). Similar trends were reported in *Hazo* [11], *Gari* (a Nigerian fermented cassava grit) [18], watermelon [19], and batter of pigeon pea grain flour [20]. But the reverse was observed in mahogany bean flour batter [21]. LAB fermentation is known to promote hydrolysis of macromolecules to enhance nutritional quality of flour-based fermented foods [22]. Increasing the moisture content of the *Helbat* product decreases its thickness to be consumed enjoyably.

**Table 6:** Proximate chemical composition of fermenting *Megulat Helbat*.

Proximate analysis	Mean (±SD) proximate composition (%)			
	Day 1	Day 3	Day 5	Day 7
Moisture content	7.03 ± 0.03^d^	8.86 ± 0.03^c^	9.17 ± 0.03^b^	11.02 ± 0.05^a^
Ash content	13.62 ± 0.82^a^	12.30 ± 0.30^b^	11.04 ± 0.23^c^	9.30 ± 0.30^d^
Crude fat	20.46 ± 0.02^d^	22.56 ± 0.40^c^	23.55 ± 0.05^c^	24.93 ± 0.05^a^
Crude protein	26.24 ± 0.12^d^	27.39 ± 0.09^c^	28.49 ± 0.06^b^	29.31 ± 0.06^a^
Crude fiber	7.33 ± 0.76^d^	9.16 ± 0.28^c^	11.25 ± 0.25^b^	11.85 ± 0.13^a^
Total carbohydrate	25.30 ± 0.08^a^	19.78 ± 0.65^b^	16.49 ± 0.16^c^	13.57 ± 0.26^d^
Energy value (Kcal/100 g)	390.40 ± 0.38^a^	391.59 ± 1.00^a^	391.89 ± 1.00^a^	396.07 ± 1.30^a^

Means (±SD) in the same row with different letters are statistically significantly different at p ≤ 0.05.

The ash content of the *Helbat* product, representing its mineral contents, has shown significant decrement as the days of fermentation progressed. Similar observations were reported in fermentation of *Hazo* [11], cocoyam flour batter [23], epicarp of watermelon [19], and sorghum, cowpea, and cowpea-sorghum blends [24]. On the other hand, while the ash content of sesame condiment was increased from 4.41 to 5.83% in four days [25], that of *Siljo* remained 7% during seven days of fermentation [6]. The decrease in ash content during fermentation might be due to cumulative effects of the spice additives in the *Helbat* metabolism and consumption of the minerals by fermenting microorganisms in the process. This observation is vital to shorten the fermentation time and avoid the depletion of the minerals or to take other mineral-rich supplements.

Fermentation has promoted the crude fat content of the *Helbat* condiment. The mean percentage crude fat content increased from 20.46 ± 0.02 (day 1) to 24.93 ± 0.05 (day 7) (*p* ≤ 0.05). This finding agrees with the observations on *Siljo*, that increased from 21 to 25% [6], *Hazo*, that increased from 4.19 to 4.74 [11], epicarp of watermelon, that increased from 6.19% to 6.25% [19], and mahogany bean batter, that increased from 0.48 to 0.67% [21]. On the other hand, the mean percentage crude fat content of *Gari* [18] and fermented pigeon pea product have decreased [20]. Since fasting prevents consumers from having fat-rich foods, consuming *Helbat* can be regarded as good supplement.

Beans are sources of protein-rich staple foods in many traditions that complement meat products. Proximate analysis showed that the mean percentage protein content increased moderately from 26.24 ± 0.12 (day 1) to 29.31 ± 0.06 (day 7). This observation agrees with the findings of Mehari and Ashenafi (1995) [6] and Gebrelibanose (2015) [11]. Whereas the first reported ca. 2% increase in *Siljo* in four days fermentation, the later reported ca. 1.6% increase in *Hazo* in six days fermentation. A study done by Igbabul et al. (2014) also reported about 4.5% increase in mean percentage composition of crude protein in mahogany flour batter after 3 days of fermentation [21]. Studies on *Awaze* [15] and *Kawal* (fermented food of Chad prepared from cassava leaves) [26] reported decrease in crude protein after fermentation. The decrease in mean percentage protein content of *Kawal* was 36%. The maintenance or increase in its protein content makes *Helbat* a good choice for fasting seasons.

Crude fiber is an important nutritional component that facilitates digestion and eases bowl movement. According to the study done by Eromosele and Eromosele (1993) high fiber content in foods expands the inside wall of the colon, easing the passage of waste, thus making it an effective anti-constipation constituent [27]. It was also established that high fiber content in foods lowers cholesterol level in blood, reduces the risk of various cancers and bowel disease, and improves the general health conditions and well-being [19]. The mean percentage of crude fiber content of *Helbat* has increased from 7.33 ± 0.76 (on day 1) to 11.85 ± 0.13 (on day 7) during fermentation. This observation agrees with the findings of other researchers who observed increase in fiber content from 6.00 to 9.26% and 2.6–4.00% in 4–5 days fermentation [19,28].

The mean percentage of total carbohydrate decreased from 25.30 ± 0.08 (on day 1) to 13.57 ± 0.26 (on day 7)–nearly half of the total carbohydrate is lost in seven days. This is a common phenomenon and many researchers have reported similar trends in *Siljo, Hazo*, banana must, millet-soya bean blends, epicarp of watermelon, *acha*, and pear millet flour fermentations [6,11,17,26,29,30]. The decrease in mean total carbohydrate content is attributed to the changing micro-environment that becomes suitable for LAB thriving on carbohydrates as their carbon sources [26]. The carbohydrate content of *Bulla*, an Ethiopian indigenous food prepared from the root of false banana-locally called *Enset* (Amharic) or *Gunaguna* (Tigrinya)-increased with fermentation period [31]. Carbohydrates are the most common dietary components of the communities that consume *Helbat*. *Helbat* is served as condiment or non-spicy, cold souse with *Tihlo* and *Injera* (two important traditional foods in eastern Tigrai). Since *Tihlo* and *Injera* are principal sources of carbohydrate, the limitation of *Helbat* in providing carbohydrates cannot be a concern. In fact, the LAB consumed with the *Helbat* can have beneficial effects [6,11].

#### 3.3.3 Mineral composition

Food processing procedures including fermentation are known to reduce the mineral contents of the foods [32]. The proximate analysis has revealed that the ash content of *Helbat* has decreased as fermentation progressed. There could be some physicochemical or biochemical mechanisms that affect the bioavailability of the minerals. But the amounts of the minerals in the 7-days fermented *Helbat* were quite high (Table 7, Row 2) to qualify as the source of the minerals.

**Table 7:** Mineral composition of *Megulat Helbat* during fermentation and storage period.

Minerals	Mean (±SD) mineral content (mg/L)			
	Fermentation	Storage period		
	Week 1	Week 3	Week 6	Week 8
Magnesium	30.60 ± 0.05^d^	50.00 ± 0.00^c^	76.60 ± 0.57^b^	112.60 ± 0.15^a^
Calcium	661.00 ± 0.10^a^	210.00 ± 0.00^b^	193.30 ± 0.57^c^	180.00 ± 1.00^d^
Potassium	1410.00 ± 0.00^a^	1017.00 ± 0.82^b^	587.30 ± 0.05^c^	186.60 ± 0.05^d^
Sodium	2114.00 ± 0.10^a^	425.30 ± 0.01^b^	371.90 ± 0.01^c^	333.30 ± 0.36^d^
Phosphorus	175.30 ± 0.05^a^	121.10 ± 0.00^b^	92.10 ± 0.01^c^	81.00 ± 0.01^d^
Nitrogen	3123.00 ± 0.10^a^	1070.80 ± 0.38^b^	377.60 ± 0.11^c^	170.80 ± 0.38^d^
Zinc	5.00 ± 0.00^d^	8.00 ± 0.05^c^	9.20 ± 0.00^b^	11.20 ± 0.00^a^
Iron	47.80 ± 0.00^a^	42.10 ± 0.22^b^	37.80 ± 0.00^c^	31.60 ± 0.00^d^
Copper	6.30 ± 0.00^a^	2.60 ± 0.03^b^	2.50 ± 0.00^b^	2.30 ± 0.00^b^

Means (±SD) in the same column with different letters are statistically significantly different at p ≤ 0.05.

#### 3.3.4 Microbial properties

Studies of the microbial property of the *Helbat* product revealed that it has LAB, *Bacillus* spp., coliforms, yeasts, and molds. The loads of these microorganisms vary from day to day throughout the fermentation period and beyond (Table 8). The load of all detected microorganisms showed increments up to the third to sixth days of fermentations and started to drop except for the molds. The rising of the LAB count during the first few days of fermentation was also observed in other Ethiopian traditional foods, condiments, and beverages like *Hazo*, *Siljo*, and *Borde* [6,11,12]. *Bacillus* spp. were also common in Ethiopian traditional condiments and beverages [6,33]. The total coliform count has also showed steady increment up to day 6. A study done by Mehari and Ashenafi observed the same trend in *Siljo* [6] while studies by others reported the reverse in *Datta* and *Hazo* [11,15]. The yeast count quickly increased in the first three days and started dropping very quickly. Similar trend was observed with yeast in *Hazo* [11]. The increase in yeast count in *Awaze* was slow, taking 14 days to reach the maximum count of 6.40 CFU/g. But no yeast growth was detected in *Datta* throughout the fermentation period [15]. Molds were detected in day 2 of fermentation and kept increasing gently. Whereas a study done by Mehari and Ashenafi observed increment in mold count till the 6th day of fermentation of *Siljo* [6], Gebrelibanose observed the lowering of mold count in *Hazo* [11]. Anyways the microbial counts during the fermentation and storage periods were far below the safe limit of 1 × 10^6^ CFU/g [34]. We believe that increasing the aseptic conditions of its preparation and storage can further reduce the coliform and mold count-via cleaning or sterilizing the utensils and ingredients as well as enhancing the cleanliness of the persons preparing the product.

**Table 8:** Microbial load of *Megulat Helbat* during fermentation and storage.

Fermentation/storage time	Mean (±SD) of microbial count (CFU/g)				
	LAB	*Bacillus* spp.	Total coliform	Yeast	Mold
Day 0	1.99 ± 0.33^g^	1.66 ± 0.33^h^	2.22 ± 0.19^gf^	3.66 ± 1.52^d^	0.00 ± 0.00^f^
Day 1	2.77 ± 0.19^gf^	2.33 ± 0.33^hg^	2.55 ± 0.38^f^	6.99 ± 0.33^c^	0.00 ± 0.00^f^
Day 2	3.55 ± 0.50^f^	3.44 ± 0.19^f^	4.11 ± 0.19^e^	8.66 ± 0.33^b^	1.22 ± 0.50^fe^
Day 3	7.77 ± 0.50^d^	5.55 ± 0.19^e^	4.44 ± 0.19^e^	10.99 ± 0.66^a^	1.55 ± 0.19^fe^
Day 4	10.88 ± 1.17^c^	6.44 ± 0.38^d^	5.55 ± 0.19^d^	6.88 ± 0.50^c^	2.33 ± 0.33^e^
Day 5	12.23 ± 0.38^b^	7.33 ± 0.57^c^	7.77 ± 0.38^b^	4.33 ± 0.33^d^	5.99 ± 0.66^d^
Day 6	15.33 ± 0.33^a^	10.88 ± 1.01^a^	9.21 ± 0.50^a^	2.33 ± 0.33^e^	6.99 ± 0.33^d^
Day 7	13.78 ± 0.96^b^	8.77 ± 0.69^b^	6.88 ± 0.83^c^	1.99 ± 0.33^f^	9.44 ± 0.50^c^
Day 15	10.88 ± 1.64^c^	6.44 ± 0.38^d^	4.77 ± 0.50^e^	1.33 ± 0.33^fe^	11.55 ± 0.50^b^
Day 30	7.33 ± 0.33^d^	4.99 ± 0.33^e^	2.44 ± 0.50^f^	1.21 ± 0.50^f^	12.55 ± 2.14^ba^
Day 45	4.93 ± 0.43^e^	2.99 ± 0.33^g^	1.77 ± 0.19^g^	0.00 ± 0.00^g^	13.21 ± 0.50^a^
Day 55	3.22 ± 0.38^f^	1.77 ± 0.19^h^	0.99 ± 0.33^h^	0.00 ± 0.00^g^	9.88 ± 1.64^c^

Means (±SD) in the same column with different letters are statistically significantly different at p ≤ 0.05.

LAB and *Bacillus* spp. are important components of fermented foods, especially condiments. Therefore, isolation and characterization of the LAB and *Bacillus* spp. were carried out through standard cellular and biochemical techniques [11,13,35,36]. This exercise helped in isolating, characterizing and plausibly identifying three strains (Table 9) – namely *Bacillus*, *Lactobacillus*, and *Pediococcus*. Whereas *Lactobacillus* dominated 75% of the samples, *Pediococcus* dominated 25% of the samples. Many researchers have observed *Lactobacillus* as the dominant LAB in fermenting traditional foods such as *Siljo*, *Hazo*, *Fufu*, *Ogi*, *Akamu*, and *Kunu*-*Zaki* [6,11,[36], [37], [38]]. The fermentation of traditional foods generally depends on naturally occurring LAB [6,11]. The LAB became dominant towards the 6th day of fermentation. They are well-known for producing a variety of inhibitory metabolic end-products such as lactic acid, acetic acid, CO_2_, H_2_O_2_, antifungal peptides, and bacteriocins that inhibit the growth of spoiling microbes.

**Table 9:** Isolation and characterization of LAB and *Bacillus* isolates from *Megulat Helbat*.

SN	Cellular morphology	Gram reaction	Motility test	Oxidase test	Catalase test	Growth at 4 °C	Growth at 45 °C	H_2_S production	Homo/hetero fermentative	Arrangement	Glucose	Mannitol	Maltose	Sucrose	Fructose	Urase	Probable bacteria
1	Rod	+	−	−	−	+	−	+	HM	S	+	+	+	+	+	+	*Lactobacillus* spp.
2	Cocci	+	−	−	−	+	+	−	HM	C	+	−	−	+	−	−	*Pediococcus* spp.
3	Cocci	+	−	−	+	ND	ND	−	ND	S	+	+	−	+	+	−	*Bacillus* spp.

+ = Positive test; − = Negative test; HM = Homo-fermentative; S = Single; C: Chain; ND = not done.

### 3.4 Characteristics of stored *Megulat Helbat*

According to the Ethiopian Orthodox Christian law, adult followers are obliged to fast. The fasting involves preventing oneself from consuming meat, dairy products, and eggs. Therefore, fasting foods, additives, and condiments like *Helbat* can be prepared during non-fasting season and kept for several weeks until the fasting season comes. This study has explored into the physicochemical and microbiological characteristics of stored *Megulat Helbat* product. This exercise can also help us establish the shelf-life of the product.

#### 3.4.1 Storage temperature and physicochemical properties

Storage temperature is a key environmental factor that affects the quality of foods by affecting some physicochemical and biochemical processes. Thus, decisions in regard to storage, transport, and packaging of food products are made based on data about the effects of temperature. Below, how varying storage temperature affects the physicochemical characteristics (namely, pH, TAA and TSS) of *Helbat* is presented.

The effects fermentation temperature was explored at four fermentation temperature ranges (<10 °C (1), 11–15 °C (2), 16–20 °C (3), and 21–25 °C (4)) during eight weeks of storage. We have observed above that the mean temperature of fermenting *Helbat* decreased with fermentation time. Likewise, the mean pH decreased with fermentation time and positively correlated with changes in mean temperature (Table 5). Similarly, the decreases in mean pH in the eight weeks of storage time at all storage temperatures were mostly statistically significant. Increasing the storage temperature has led to decrease in mean pH throughout the storage time (Table 10). The decrease in mean pH during the seven day fermentation period was ca. 2.20 (Table 5). Interestingly, the decrease in mean pH at all storage temperature ranges between the first and the eighth weeks of storage were between 0.18 (at 21–25 °C storage temperature) and 0.30 (at 11–15 °C storage temperature) implying that change in pH during the eight of weeks storage period was very mild.

**Table 10:** Physicochemical properties of *Megulat Helbat* during the eight weeks storage.

Physicochemical Parameters	Weeks of storage	Mean (±SD) values of physicochemical parameters			
		<10 °C	11–15 °C	16–20 °C	21–25 °C
pH	1	3.86 ± 0.01^a^	3.71 ± 0.01^a^	3.55 ± 0.01^a^	3.45 ± 0.00^a^
	2	3.82 ± 0.01^b^	3.68 ± 0.00^b^	3.49 ± 0.01^b^	3.41 ± 0.01^b^
	3	3.75 ± 0.00^c^	3.63 ± 0.01^c^	3.48 ± 0.01^b^	3.37 ± 0.01^c^
	4	3.71 ± 0.01^d^	3.56 ± 0.01^d^	3.43 ± 0.01^c^	3.31 ± 0.00^d^
	5	3.69 ± 0.01^e^	3.54 ± 0.01^e^	3.38 ± 0.01^d^	3.29 ± 0.00^f^
	6	3.64 ± 0.02^f^	3.47 ± 0.01^f^	3.34 ± 0.00^e^	3.29 ± 0.10^f^
	7	3.62 ± 0.01^f^	3.43 ± 0.00^g^	3.31 ± 0.00^f^	3.28 ± 0.10^g^
	8	3.58 ± 0.01^g^	3.41 ± 0.01^h^	3.30 ± 0.08^f^	3.27 ± 0.06^g^
TTA (%)	1	0.66 ± 0.01^h^	0.55 ± 0.01^h^	0.47 ± 0.01^g^	0.39 ± 0.02^g^
	2	0.73 ± 0.00^g^	0.64 ± 0.01^g^	0.51 ± 0.00^f^	0.41 ± 0.00^g^
	3	0.76 ± 0.01^f^	0.68 ± 0.01^f^	0.52 ± 0.00^f^	0.50 ± 0.01^f^
	4	0.81 ± 0.01^e^	0.71 ± 0.02^e^	0.57 ± 0.01^e^	0.58 ± 0.01^e^
	5	0.83 ± 0.01^d^	0.74 ± 0.01^d^	0.60 ± 0.01^d^	0.62 ± 0.02^d^
	6	0.88 ± 0.00^c^	0.78 ± 0.01^c^	0.66 ± 0.01^c^	0.67 ± 0.00^c^
	7	0.93 ± 0.00^b^	0.84 ± 0.01^b^	0.71 ± 0.01^b^	0.69 ± 0.01^b^
	8	0.97 ± 0.00^a^	0.88 ± 0.01^a^	0.78 ± 0.01^a^	0.75 ± 0.01^a^
TSS (°Brix)	1	6.50 ± 0.02^a^	6.17 ± 0.02^a^	5.52 ± 0.02^a^	4.92 ± 0.02^a^
	2	6.34 ± 0.03^b^	6.00 ± 0.00^b^	5.28 ± 0.01^b^	4.88 ± 0.01^a^
	3	6.25 ± 0.02^b^	5.73 ± 0.03^c^	5.16 ± 0.04^c^	4.57 ± 0.02^b^
	4	6.23 ± 0.22^b^	5.45 ± 0.05^d^	4.88 ± 0.02^d^	4.13 ± 0.02^c^
	5	5.84 ± 0.03^c^	5.24 ± 0.04^e^	4.63 ± 0.01^e^	3.81 ± 0.02^d^
	6	5.47 ± 0.07^d^	4.86 ± 0.05^f^	4.55 ± 0.05^f^	3.48 ± 0.02^e^
	7	5.24 ± 0.05^e^	4.75 ± 0.05^g^	4.32 ± 0.02^g^	3.26 ± 0.02^f^
	8	4.94 ± 0.02^f^	4.50 ± 0.01^h^	4.15 ± 0.05^h^	2.81 ± 0.01^g^

Means (±SD) in the same column with different letters are statistically significantly different at p ≤ 0.05.

The acidity of fermented *Helbat* and other fermented foods is because of organic acids naturally present in the ingredients. The mean TTA (%) of *Megulat Helbat* increased by about 0.40% between the first and seventh days of fermentation (Table 5). Similarly, it showed statistically significant increments, in most cases, between storage weeks at all storage temperatures (Table 10). But as compared to the changes between the first and seventh days during the fermentation period (0.50%), the changes between the first and eighth weeks of storage was, relatively speaking, mild, ranging from 0.31% (in <10 °C storage temperature) to 0.36% (in 21–25 °C storage temperature). The simultaneous decrease of pH and increase of TTA during the storage weeks could be due to LAB activities that degrade the carbohydrates leading to acidification. Similar patterns were reported in *Hazo* [11] and millet-soya bean blends [17].

Total soluble solutes (TSS) (in °Brix) represents sugar and other dissolved solids in the solution. Mean TSS of *Megulat Helbat* decreased with increasing storage time and temperature-the decrease between consecutive weeks being statistically significant (Table 10). The decrease in mean TSS during the seven day fermentation period (6 °Brix) (Table 5) is much higher than the decrease during the eight weeks storage period-ranging between 1.37 °Brix (in 16–20 °C storage temperature) and 2.11 Brix (in 21–25 °C storage temperature). This lowering in TSS during the storage period could be due to the use of carbohydrates by microorganisms found in the fermented *Helbat*. Similar results were reported with the fermentation of banana must [29,30].

#### 3.4.2 Storage time and proximate composition

Data of proximate analyses showed that increments in mean percentage moisture content and mean percentage crude fiber were significant. The remaining constituents of the analyses demonstrated decrements in the eight weeks of storage. However, the changes were very mild in all cases (Table 11). This observation can have three implications. (1) With increasing storage time, the biochemical processes that bring about changes in the constituents are pretty much slowed down as the environment becomes more anaerobic. (2) The continuous lowering of total carbohydrate content and the corresponding increment of crude fiber may be due to activities of LAB as established in many other research reports in *Siljo, Hazo*, banana must, millet-soya bean blends, and epicarp of water melon [6,11,17,19,29,30]. Their presence in the *Helbat* product can have useful effects on the intestinal microbiome of consumers. (3) Increasing storage time depletes nutritionally useful constituents for fasting consumers (especially the fat and protein contents). Therefore, storage of *Helbat* for longer time might not be recommended for consumers who cannot obtain protein and fat supplements from other sources.

**Table 11:** Proximate chemical composition of stored *Megulat Helbat*.

Proximate analysis	Mean (±SD) of proximate composition (%)			
	Week 1	Week 3	Week 6	Week 8
Moisture content	11.52 ± 0.05^d^	13.93 ± 0.02^c^	16.17 ± 0.02^b^	17.12 ± 0.02^a^
Ash content	9.30 ± 0.30^a^	8.46 ± 0.03^b^	8.06 ± 0.03^c^	7.29 ± 0.03^d^
Crude fat	24.95 ± 0.05^a^	21.85 ± 0.05^b^	20.55 ± 0.05^c^	19.98 ± 0.00^d^
Crude protein	29.31 ± 0.06^b^	29.74 ± 0.06^a^	27.51 ± 0.20^c^	25.80 ± 0.12^c^
Crude fiber	11.85 ± 0.13^d^	13.08 ± 0.38^c^	15.57 ± 0.25^b^	18.80 ± 0.05^a^
Total carbohydrate	13.07 ± 0.26^a^	12.85 ± 0.36^a^	12.69 ± 0.19^a^	10.22 ± 0.04^b^
Energy value (Kcal/100 g)	394.07 ± 1.30^a^	367.06 ± 2.13^b^	345.78 ± 0.28^c^	323.95 ± 0.41^d^

Means (±SD) in the same row with different letters are statistically significantly different at p ≤ 0.05.

#### 3.4.3 Storage time and mineral composition

Determination of the ash content using the proximate analysis has showed a mild decrement. Mineral analysis of the product in the eight weeks of storage revealed that all tested minerals except Mg and Zn showed drastic decrements (Table 7). This finding clearly implies that stored *Helbat* is not good source of the tested minerals. This calls for seeking alternative sources for mineral supplements or modification of the preparation and storage of the condiment. Further investigation on how the storage conditions and the *Helbat* microflora affect the bioavailability of minerals can be recommended.

#### 3.4.4 Storage time and microbial properties

Storage of the *Helbat* product up to 55 days revealed that the loads of the tested microbes were decreasing with storage time, with the exception of molds (Table 8). In all cases, the decrease in mean microbial count (CFU/g) between two consecutive testing days was statistically significant (*p* ≤ 0.05). But the mean microbial count of molds was rising until day 45. Relatively speaking, the mean microbial counts of the LAB and *Bacillus* spp. were higher than that of the coliforms and yeasts.

### 3.5 Shelf life evaluation of *Megulat Helbat*

The shelf life of *Megulat Helbat* was tested at different storage temperature ranges, indicated in the preceding sections, by evaluating: (a) its overall acceptability using sensory evaluation and (b) its ability to inhibit the growth of spoiling microbes.

#### 3.5.1 Overall acceptability

The sensory evaluation was carried out by a panel of 30 participants who consume *Helbat* regularly. The products stored at the first, second, third, fourth storage temperatures, and the control were evaluated three, four, three, one, and one times, respectively. *Helbat* stored at the second storage temperature (11–15 °C) received significantly the highest mean rating by the 55th day of storage (*p* ≤ 0.05; Table 12). This implies that *Helbat* can be kept up to 40–55 day if stored at <20 °C. Note that *Helbat* stored at ambient temperature (control) has received the lowest mean sensory evaluation ratings, implying that storing the product at uncontrolled high temperatures causes the loss of its sensory qualities.

**Table 12:** Results of sensory evaluation of stored *Megulat Helbat*.

Storage temperature (°C)	Number of Evaluations	Mean (±SD) scores of sensory attributes (Degrees of likings)					Shelf life (in days)
		Taste	Color	Aroma	Mouth feel	Overall acceptability	
1: <10	3	6.33 ± 0.33^b^	7.55 ± 0.19^b^	7.44 ± 0.38^b^	7.22 ± 0.19^b^	6.66 ± 0.33^b^	40
2: 11–15	4	8.83 ± 0.19^a^	8.22 ± 0.38^a^	9.00 ± 0.00^a^	8.22 ± 0.19^a^	7.55 ± 0.19^a^	55
3: 16–20	3	6.33 ± 0.33^b^	5.33 ± 0.33^c^	6.33 ± 0.33^c^	7.22 ± 0.38^b^	7.44 ± 0.19^a^	42
4: 21–25	1	4.44 ± 0.38^c^	4.22 ± 0.38^d^	5.00 ± 0.00^d^	5.55 ± 0.19^c^	6.22 ± 0.19^b^	12
5: Control	1	3.33 ± 0.33^d^	4.33 ± 0.33^d^	5.11 ± 0.19^d^	4.11 ± 0.19^d^	4.33 ± 0.33^c^	10

Means (±SD) in the same column with different letters are statistically significantly different at p ≤ 0.05.

#### 3.5.2 Inhibiting growth of spoiling microbes

Lowering storage temperature is the most recommended procedure to store food and other perishable products for longer period. It is a textbook knowledge that with increasing storage temperature, the likelihood that spoiling microbes grow is very high. In the present study, the second storage temperature (11–15 °C) inhibits the growth of spoiling microbes better than lower or higher storage temperatures (Table 13). Logically speaking, the storage temperature that would have been the most effective in inhibiting the growth of spoiling microbes is the first one (<10 °C). This observation (that may be described as anomaly) may be described by some physicochemical, biochemical, or microbial characteristics of the product that can inhibit the growth of spoiling microbes. The highest shelf life achieved at the second storage temperature can be attributed to suitable physicochemical, biochemical, or microbial processes created by that storage temperature. But generally speaking, <20 °C storage temperatures have kept the product safe.

**Table 13:** Observation of spoiling yeasts and molds as a function storage temperature and time.

Storage temperature (°C)	Date of observation for the first visible yeast and mold growth	Shelf life (in days)
1: <10	No yeast and mold growth observed until day 49	49
2: 11–15	No yeast and mold growth observed until day 56	56
3: 16–20	No yeast and mold growth observed until day 42	42
4: 21–25	No yeast and mold growth observed until day 12	12
Control	No yeast and mold growth observed until day 9	9

## 4 Conclusion

Food processing, including fermentation, changes the quality of foods in some ways. This study has helped us identify the best variety and suggest the best formulation to produce the best *Helbat*. We have seen how fermentation and storage time as well as storage temperature affect the physicochemical characteristics, proximate and mineral compositions, microbial load, and sensory quality of the *Helbat* product like other traditional condiments in Ethiopia and elsewhere. The information gained is helpful in deciding the proportion of the ingredients in preparing the best *Helbat* and the fermentation and storage durations and temperature ranges that yield high quality product. The study has also shown that *Helbat* can be stored for more than eight weeks without losing its sensory quality and spoilage. However, the longer it is stored, the more the depletion of its nutrients. Thus, it is correct to suggest that *Helbat* should be consumed fresh (i.e., within 7 days) during the fasting seasons. But further research may be required to conclusively recommend that. Finally, exploring into the antioxidant properties of the condiment and its LAB may highlight its qualities further.

## Author contribution statement

Solomon Fitsum: Conceived and designed the experiments; Performed the experiments; Analyzed and interpreted the data; Wrote the paper.

Goitom Gebreyohannes Berhe, Gebreselema Gebreyohannes: Conceived and designed the experiments; Analyzed and interpreted the data; Contributed reagents, materials, analysis tools or data.

Desta Berhe Sbhatu: Conceived and designed the experiments; Analyzed and interpreted the data; Wrote the paper.

## Data availability statement

Data will be made available on request.

## Declaration of competing interest

The authors declare that they have no known competing financial interests or personal relationships that could have appeared to influence the work reported in this paper.
